# Evaluation of treatment adherence in patients with mental illness

**DOI:** 10.1192/j.eurpsy.2022.1598

**Published:** 2022-09-01

**Authors:** H. Jemli, R. Jomli, H. Ghabi, U. Ouali, M. Ben Amor, Y. Zgueb

**Affiliations:** 1 university of tunis elmanar, Faculty Of Medicine Of Tunis, manouba, Tunisia; 2 Razi Hospital, Psychiatry A, manouba, Tunisia

**Keywords:** Adherence, patient, Treatment, mental disorder

## Abstract

**Introduction:**

Treatment adherence, is defined as “the extent to which a person’s behavior — taking medication, following a diet, and/or executing lifestyle changes — corresponds with the agreed recommendations from a healthcare provider.” The course of patients with mental health is habitually chronic and based on an indefinite continuation of treatment to sustain remission and prevent relapses. Treatment adherence issues are the main obstacles in the management of these patients

**Objectives:**

The aim of the present study was to evaluate treatment adherence in patients with mental health and the demogrphic and clinical factors associated with it.

**Methods:**

It was a cross-sectional study conduced at the department of Psychiatry A at Razi Hospital.The validated arabic version of Morisky-Green test was used to assess medication adherence. The patients were considered as adherent if they answered ‘No ’ to all questions

**Results:**

60 patients were included, with a sex ratio M / F of 0.47. Patients were treated for bipolar disorder type1 in 45% of cases, schizophrenia in 28.3% of cases, schizoaffective disorder in 10% of cases and depressive disorder in 6.7% of cases. 50% of included patients had Moderate level of adherence, 35% were considered as non- adherent and only 13.3% had high adherence. The reported reasons for treatment discontinuation were insight (50%), financial problems (26.9%), side effects (15.4%) and unavailability of drugs (7.7%). The Morisky-Green test score were not correlated neither to the nature of the psychiatric disorder nor to multiple medication.

**Conclusions:**

We found a high proportion of nonadherence in patients with mental illness.

**Disclosure:**

No significant relationships.

